# A Comprehensive Review on Natural Bioactive Compounds and Probiotics as Potential Therapeutics in Food Allergy Treatment

**DOI:** 10.3389/fimmu.2020.00996

**Published:** 2020-05-22

**Authors:** Kunal Pratap, Aya C. Taki, Elecia B. Johnston, Andreas L. Lopata, Sandip D. Kamath

**Affiliations:** ^1^Molecular Allergy Research Laboratory, Discipline of Molecular and Cell Biology, College of Public Health, Medical and Veterinary Sciences, James Cook University, Townsville, QLD, Australia; ^2^Australian Institute of Tropical Health and Medicine, James Cook University, Townsville, QLD, Australia; ^3^Center for Molecular Therapeutics, James Cook University, Townsville, QLD, Australia; ^4^Faculty of Veterinary and Agricultural Sciences, The University of Melbourne, Parkville, VIC, Australia

**Keywords:** food allergy, gut microbiome, immunotherapy, medicinal plants, natural compounds, polysaccharides, probiotics, allergen

## Abstract

Food allergy is rising at an alarming rate and is a major public health concern. Globally, food allergy affects over 500 million people, often starting in early childhood and increasingly reported in adults. Commercially, only one approved oral immunotherapy-based treatment is currently available and other allergen-based immunotherapeutic are being investigated in clinical studies. As an alternative approach, a substantial amount of research has been conducted on natural compounds and probiotics, focusing on the immune modes of action, and therapeutic uses of such sources to tackle various immune-related diseases. Food allergy is primarily mediated by IgE antibodies and the suppression of allergic symptoms seems to be mostly modulated through a reduction of allergen-specific IgE antibodies, upregulation of blocking IgG, and downregulation of effector cell activation (e.g., mast cells) or expression of T-helper 2 (Th-2) cytokines. A wide variety of investigations conducted in small animal models or cell-based systems have reported on the efficacy of natural bioactive compounds and probiotics as potential anti-allergic therapeutics. However, very few lead compounds, unlike anti-cancer and anti-microbial applications, have been selected for clinical trials in the treatment of food allergies. Natural products or probiotic-based approaches appear to reduce the symptoms and/or target specific pathways independent of the implicated food allergen. This broad range therapeutic approach essentially provides a major advantage as several different types of food allergens can be targeted with one approach and potentially associated with a lower cost of development. This review provides a brief overview of the immune mechanisms underlying food allergy and allergen-specific immunotherapy, followed by a comprehensive collection of current studies conducted to investigate the therapeutic applications of natural compounds and probiotics, including discussions of their mode of action and immunological aspects of their disease-modifying capabilities.

## Introduction

Food allergy is a type-I hypersensitivity reaction caused by protein antigens found in various food sources, marked by elevated levels of IgE antibodies that can lead to potentially life-threatening clinical reactions. Allergic diseases are a global health issue posing a significant social and economic burden and reducing the quality of life (1, 2). Every year food allergy alone costs more than USD 24 billion to the US economy, and a recent systematic review estimated a much higher economic burden at the household-level (2, 3). Prevalence-based studies have reported an alarming increase in food allergy in recent years, especially among children, reaching as high as 10% (1, 4, 5). For example in Europe and the United States cases of food allergy have been reported in 8–11% of the children and adult population (6–10). More than 90% of all allergic episodes are recorded against eight major food groups: peanut, tree nuts, milk, wheat, soy, egg, fish and shellfish (1, 4, 10, 11). In a recent cross-sectional survey involving over 40,000 adults in the US, it was shown that at least 10% are food allergic, with the most common food allergy being to shellfish followed by milk, peanut, tree nut, and fish (10). Currently strict and careful avoidance of the offending food item is considered the best approach for preventing accidental allergic reactions (5). In case of accidental exposure and subsequent severe reaction, an epinephrine auto-injector (EpiPen) is the only life-saving option (5).

Naturally occurring bioactive compounds have been extensively investigated in recent years for their immunomodulatory properties with the therapeutic potential to treat various human diseases including asthma, diabetes, and cancer (12–14). Bioactive compound sources such as marine algae, Chinese herbal medicine and traditional herbal medicines have been reported to be beneficial in modulating allergic responses (15–17). The natural abundance of different types of marine algae and herbal sources provides an array of options, making them a prime source for natural-product based therapeutic approaches (18–20).

The gut microbiome may play a significant role in regulating the physiological, immunological and structural changes in the gut (21–25). Gut microbiome dysbiosis is reported to influence an array of chronic conditions including, asthma, autoimmune diseases, and food allergy (23, 26). Probiotics in general have therefore emerged as potential alternative therapeutics in the past decade. Beneficial probiotic bacteria were used to modulate the immune response through targeting Th-1, Th-2, Th-17, regulatory T (Treg) cells and B cells (23, 26, 27). The microbiota regulates all facets of the development of tolerance to food proteins during the early stages of life (28). The identification and characterization of the protective bacterial taxa and their metabolites can assist in the development of possible therapeutic approaches for food allergy by modulating the pathogenesis of allergic diseases (27–30).

Active compounds isolated from natural sources, as well as probiotics, have found immense applications in improving human health and well-being. Over the past few decades, natural products have been used to treat or alleviate symptoms for various human disorders, including immune-related diseases. In recent years, there has been a concerted effort to develop curative treatment solutions for allergic diseases, particularly for food allergy.

In this review, we provide a detailed insight into the current research developments on natural bioactive compounds and probiotics as potential candidates for the prevention and treatment of food allergy. We discuss how alternate approaches based on sources such as marine algae and traditional Chinese medicine (TCM) assist in modulating and alleviating IgE mediated allergic responses in food allergy. We then describe probiotics that help promote intestinal immunity by altering the composition of the intestinal microbiota, changing the phenotype and functions of immune cells. By providing a brief overview of the immune pathways involved in food allergy and their modulation by natural bioactive compounds or probiotics, we attempt to summarize our current understanding of the underlying modes of action. We further discuss the pitfalls and future perspectives on this approach to prevent or treat food allergy.

## Mechanisms of Allergic Reactions and Current Immunotherapeutic Approaches for Food Allergy

Food allergy is an acute hypersensitivity reaction, triggered by IgE antibodies generated against specific food allergens. A hypersensitivity reaction can lead to systemic or local inflammatory responses, resulting in swelling, urticaria, eczema, airway hyper-responsiveness, asthma, and a life-threatening severe systemic response such as anaphylaxis.

**Figure 1:**
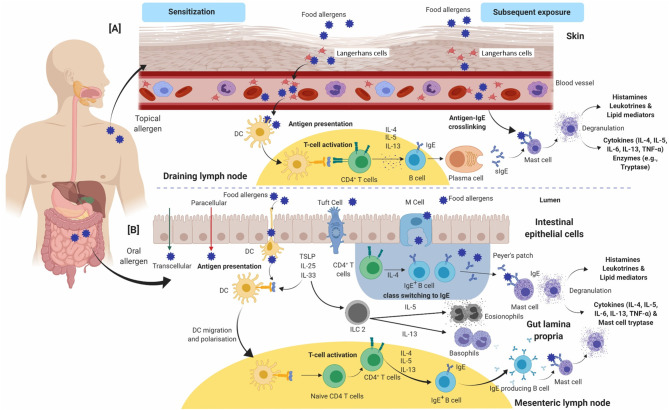
An overview of the immunological events occurring during allergic sensitization and effector phase upon exposure to food allergens via **(A)** skin and **(B)** gut. **(A)** In the epidermis, allergens are sampled by Langerhans cells and the adaptive immune response is developed in draining lymph nodes. **(B)** In the gut lumen, allergens are taken up by DCs and the subsequent events take place in Peyer's patches/mesenteric lymph nodes. Antigen-presenting cells (Langerhans cells in **A** or dendritic cells in **B**) present allergen-derived peptides to naïve CD4^+^ T-cells via MHC-class II complex. In healthy individuals, a tolerogenic immune response develops, mediated by regulatory T-cells and IL-10. In susceptible individuals, naïve CD4^+^ T-cells polarize toward a Th-2 phenotype and produce IL-4, IL-5, and IL-13. IL-4 and IL-13 induce production of allergen specific IgE antibody by B cells and clonal expansion. Allergen-specific IgE binds to FcεRI receptors on the surface of basophils and mast cells. This entire process is called allergic sensitization. On subsequent exposure to the same allergen (via contact or ingestion), the allergens bind and crosslink cell-bound IgE antibodies, which triggers degranulation and release of chemical mediators such as histamine, cytokines and prostaglandins. These mediators are responsible for the manifestation of an allergic reaction. Cytokines including IL-4, IL-5, IL-6, IL-13, and TNF-α are further released which leads to cell-mediated late-phase allergic reactions through recruitment of eosinophils and Th-2-cells. CD, Cluster of differentiation; DC's, Dendritic cells; FcεRI, High affinity immunoglobulin E receptor; MHC, Major histocompatibility complex; Th-2, T-helper-2; IL, Interleukin; IFN-γ, Interferon γ; TNF-α, Tumor necrosis factor-α.

A comprehensive overview of the mechanism of a typical type-I hypersensitivity reaction is presented in Figure 1. The sequences of events in the development of an allergic reaction begins with allergen presentation to the immune system via the gastrointestinal system, respiratory tract, or the skin, which leads to allergen specific IgE antibody production. This phase is termed “allergic sensitization” (Figure 1). IgE-dependent food allergies often manifest in infancy or early childhood, however, adult-onset food-allergies cases are increasingly recorded (10, 31, 32). Mononuclear phagocytes in the gut and the Langerhans cells in the skin are central to the translocation of food allergens across epithelial barriers. The sensitization phase involves allergen presentation to naïve CD4^+^ T cells by antigen-presenting cells (APC) such as dendritic cells (DCs), resulting in the activation and differentiation of T cells into typically CD4^+^ T cells. In response, activated Th-2 cells release cytokines, including interleukin-4 (IL-4), IL-5, and IL-13, which can promote the immunoglobulin class switch in B cells and differentiation into IgE secreting plasma cells (Figure 1). The secreted antibodies bind to the FcεRI receptor on the surface of mast cells and basophils through its Fc region. Subsequent exposure to identical or similar allergens leads to binding and cross-linking of two or more cell surface bound IgE antibodies. Allergen-induced IgE crosslinking triggers biochemical signals, leading to cell degranulation, synthesis and secretion of lipid mediators, as well as the release of Th-2 promoting cytokines (Figure 1) (33–36). The major mediators released during degranulation include vasoactive amines, lipids, cytokines and proteases that are responsible for the manifestation of clinical symptoms of a typical allergic reaction. Histamine is an early phase mediator of an allergic reaction that causes vasodilation, an increase in vascular permeability, and contraction of smooth muscles. The release of proteases, like mast cell proteases (MCP), may cause local tissue damage contributing to inflammatory conditions including asthma (33, 34, 37). Prostaglandins and leukotrienes follow suit and have a very similar effect on smooth muscles and vascular dilation (38). Cytokines initiate the late phase reaction by recruiting leukocytes such as eosinophils, neutrophils, and Th-2 cells. Mast cell degranulation activates the release of tumor necrosis factor (TNF) and IL-4, promoting inflammation by attracting neutrophils and eosinophils in multiple sites. Eosinophils and neutrophils in turn can release proteases, which can lead to localized tissue damage (e.g., eosinophilic esophagitis) (39). Th-2 cells may exacerbate the reaction by producing IL-5, recruiting more eosinophils to tissue sites and causing tissue injury (Figure 1) (39).

Currently available treatment strategies involve allergen-specific immunotherapy (AIT) which is a specialized and targeted treatment procedure performed to induce tolerance in individuals against specific food allergens (40). AIT exposes the allergic individual to small but increasing doses of the allergenic protein, resulting in desensitization or lowered allergen reactivity. The main goal of AIT is to achieve sustained immune unresponsiveness to the food allergen (5, 40, 41). There are different routes by which AIT can be administered such as SCIT (subcutaneous immunotherapy), SLIT (sublingual immunotherapy), OIT (Oral Immunotherapy), IDIT (intradermal immunotherapy), EPIT (epicutaneous immunotherapy) LNIT (local nasal immunotherapy) and ILIT (intralymphatic immunotherapy) depending on the types of allergens (40, 42). OIT is currently one of the preferred ways of administering AIT for peanut, egg and milk allergy, and has been reported to induce desensitization (43). Recently, the Food and Drug Administration (FDA, USA) approved PALFORZIA [Peanut (*Arachis hypogaea*) Allergen Powder-dnfp, Aimmune Therapeutics] as an OIT and the first therapeutic available for the treatment of peanut allergy for patients aged 4 years through 17 years of age (44). However, for other food allergen sources there are currently no curative therapies available. AIT is currently the most researched and potentially therapeutic approach for food allergy, which has a disease-modifying capacity.

**Figure 2:**
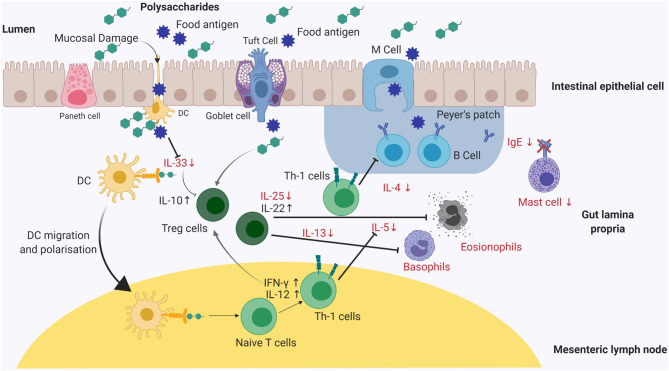
A graphical summary of the effects on different cell populations and cytokines, involved in an allergic immune response, after exposure to polysaccharides, herbal medicinal plants or traditional Chinese medicine. The reported mode of action of either natural polysaccharides or medicinal plants appears to be quite similar in murine models. Mice supplemented with abovementioned sources demonstrates reduced allergen-specific IgE antibody responses and a marked reduction in clinical symptom severity including diarrhea and drop in temperature (clinical symptoms not shown). Reduced IgE expression levels inhibits mast cell degranulation leading to symptomatic relief. Polysaccharides and medicinal plants mediate Th-1 pathway by expressing IFN-γ, IL-10, IL-12, and IL-22 maintaining the intestinal epithelial barrier function and preventing antigen presentation to dendritic cells (DC). Reduced levels of chemokines such as IL-25 and IL-33 results in decreased antigen presentation on DC's. Th-1, T-helper-1; DC's, Dendritic cells; IL, Interleukin; IFN-γ, Interferon gamma.

**Figure 3:**
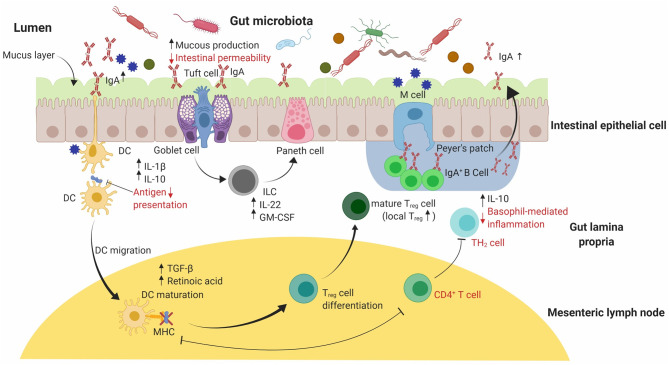
A graphical summary of the immunomodulatory effects of probiotic supplementation on the allergic response in small animal models. An equilibrated intestinal epithelial barrier in conjunction with commensal bacteria used in probiotics have been shown to protect against allergic sensitization to food allergens. Probiotic supplementation assists in maintaining the intestinal epithelial barrier's integrity by increasing mucus production through stimulated goblet cells. Release of IL-22 and GM-CSF by ILC cells in the lamina propria promotes mucus production and increased barrier function by stimulating Paneth cells to produce anti-microbial peptides (not shown). Reduced intestinal permeability and increased mucus production reduces the risk of food allergen translocation into systemic circulation. Probiotics can include a range of essential bacteria in the mix, thereby producing different metabolites responsible for elevated expression of Treg cells in the gut. An increase in Treg cells leads to the production of TGF-β and retinoic acid, favoring localized IgA antibody production by IgA^+^ B-cell antibody switching in Peyer's patches as well as increased production of IL-10 and IL-1β. Secretory IgA antibody promote systemic tolerance to food allergens by translocating into the lumen. IL, Interleukin; GM-CSF, Granulocyte-Macrophage Colony-stimulating factor; ILC, Innate lymphoid cells.

Recently Pajno et al. published EAACI guidelines on AIT for IgE mediated food allergy providing comprehensive information on the evidence-based dose recommendations for different type of AIT regimens in clinically diagnosed patients of food allergy (41). EAACI guidelines further elaborate and discuss the safe implementation of existing immunotherapy, associated inherent issues and challenges based on medicinal and social outlook (41). This review primarily focuses on the potential of natural bioactive compounds and probiotics as novel candidates in the prevention and treatment of food allergy. These sources may have the potential to complement current AIT-based approaches to provide tolerance against allergic diseases. A specific overview on the mode of action of marine algae, TCM and probiotics is discussed ahead, with particular focus on natural compounds and probiotic bacteria formulations that affect specific immunological signaling pathways (Figures 2, 3).

## Natural Compounds for Food Allergy Treatment

Natural compounds have been an attractive source for the prevention or treatment of various immunological disorders. The efficacy of natural compounds has been extensively reported in past decades. The sources for these natural compounds include polysaccharides from marine algae or non-algal origins, traditional medicinal systems such as TCM and medicinal plants. We discuss the role and efficacy of natural compounds in influencing allergic disorders, with a specific focus on food allergy.

### Polysaccharides

Polysaccharides are a heterogeneous group of macromolecules with various biological properties that can act as potential therapeutics for human diseases (45). These macromolecules consist of large monomeric units of monosaccharides joined together by glycosidic linkages. Polysaccharides can be hydrolyzed by acid hydrolysis or with the aid of specific enzymes, to produce the monomeric monosaccharide units (46, 47). Based on the type of monomeric units, the polysaccharides can be distinguished as homopolysaccharides (homoglycans) or heteropolysaccharides (heteroglycans) (48). Heteropolysaccharides may also contain non-carbohydrate units along with the monosaccharides. In the human gut, *Bacteroidetes, Firmicutes* and *Actinobacteria* are some of the dominant phyla responsible for enzymatically degrading dietary polysaccharides and producing functional secondary metabolites such as short chain fatty acids (SCFA's), and mucin (45, 49). These dominant bacterial phyla's are responsible for secreting different classes of CAZymes (carbohydrate active enzymes) such as glycan utilizing glycoside hydrolases, carbohydrate esterases, sulfatases, and polysaccharide lyases, which are further classified and reported based on family members of respective enzymes in the CAZy database (www.cazy.org) (50, 51). Conversely, the human gastrointestinal system secretes a limited number of enzymes essential for digesting dietary fiber polysaccharides such as starch. Interestingly studies have reported the ability of geographically distinct populations that can catabolize marine algal polysaccharides such as alginate, carrageenan and porphyrans (52, 53).

Different polysaccharides have been reported to contain sugar molecules, such as galactose, rhamnose, fucose, and arabinose, acting as immune potentiators; and reported for their anti-coagulant, anti-HIV and anti-oxidant activities (47, 54–57). Moreover, studies have also reported that polysaccharides can influence the immune response upon digestion by downregulating Th-2 cytokines and suppressing allergic inflammatory responses in the gut (17, 58–61). A brief summary of these investigations is provided in Table 1.

**Table 1:** A summary of studies investigating the immunomodulatory effects of active oligo- and polysaccharide components from various natural sources, tested for preventative (prophylactic) or treatment (curative) strategies, using mouse models of food allergy.

**Sr. no**.	**Source of natural bioactive compounds**	**Active component**	**Allergen source**	**Strain, route of exposure**	**Treatment strategy**	**Examined parameters**	**Outcomes**	**References**
1	*Undaria pinnatifida* (Wakame)	Mekabu fucoidan	Ovalbumin	BALB/c, NA	- NA/Preventative	- IL-4, IL-5, IL-13, IFN-γ - IgE, eosinophils in BALF - Histology (H&E)	↓ IgE producing B-cells ↓ IgE production by I.P. administering fucoidan	(62, 63)
2	*Porphyra haitanensis* (Red Algae)	Sulfated polysaccharides and R-phycocyanin	Tropomyosin	BALB/c, i.p.	- Treatment	- Anaphylactic symptom scorin - IL-4, IL-13, IFN-γ expression - Histamine in feces and sera - β-hexosaminidase and histamine by RBL-2H3 cell	↓ Anaphylactic score ↓ Histamine and TM-specific IgE ↓ IL-4, IL-13 ↑ IFN-γ	(64)
3	*Porphyra haitanensis* (Red Algae)	Sulfated polysaccharides (PHPS)	Tropomyosin	BALB/c, i.p.	- Preventative and Treatment	- TM-specific IgE, IgG1 and IgG2a in sera - Histamine in feces - Histology - IL-4, IL-5, IL-13, IL-10 and IFN-γ expression	↓ Histamine and TM-specific IgE, IgG1 ↑ IgG2a ↓ IL-4, IL-5, IL-13 ↑ lymphocytic infiltration in jejunum ↑ IFN-γ	(65)
4	*Gracilaria lemaneiformis* (Red Algae)	Sulfated polysaccharides	Tropomyosin	BALB/c, i.p.	- Preventative and Treatment	- Anaphylactic symptom scoring - MLN cell re-stimulation for IL-4, IL-13, IFN-γ, and TGF-β β-hexosaminidase and histamine by RBL-2H3 cell - Proteome Profiler - TM array for MAPK family, AKT family - and p70 S6 kinase in KU812 cells	↓ Anaphylactic score Restored temperature ↓ mMCP-1, Histamine and TM-specific IgE ↑ Foxp3 and ↓ GATA-3 ↓ IL-4, β-hexosaminidase and histamine ↓ p38 MAPK	(66)
5	Oyster-derived polysaccharides	Polysaccharides	Ovalbumin	BALB/c, i.p.	- Preventative/ Preventative and Treatment	- Symptomatic Scoring - IHC Staining (H7E) - Morphometric analysis - Mast cell expression by toluidine blue - IL-4, IFN-γ, T-bet expression levels	↑ Allergic diarrhea Restored villus/crypt ratio in duodenum ↓ Mast cell infiltration in duodenum ↓ IL-4^+^ cells in duodenum ↓ IL-4 in splenocytes ↑ IFN-γ and T-bet	(67, 68)
6	Fructo-oligosaccharides	Fructo-oligosaccharides	Ovalbumin	BALB/c [OVA23-3(+/–)], mixed in the diet	- Preventative	- OVA-specific and total IgE levels - IL-2, IL-4, IFN-γ levels - mMCP-1 levels	↓ IL-2, IL-4, IL-13 and IFN-γ ↓ mMCP-1 ↓ Total IgE	(20)
7	*Eucheuma cottonii* (Gusô)	Sulfated Oligosaccharides	Tropomyosin	BALB/c, i.p.	- Preventative and Treatment	- Symptomatic Scoring - TM-specific-IgE, IgG1 and IgG2a - Histamine levels and mMCP-1 levels - IL-4, IL-10, Il-13, and IFN-γ levels	Restored temperature ↓ Anaphylactic score ↓ Diarrhea incidences ↓ IgE and IgG1 ↑ IgG2a ↓ MCP-1 and Histamine ↓ degranulation of mast cells in intestine ↓ IL-4, L-13 ↑ IL-10 and IFN-γ	(69)
8	Aloe Vera	Processed Aloe Vera Gel polysaccharides	Ovalbumin	BALB/c, i.p.	- Preventative	- Symptomatic Scoring - OVA-specific-IgE, IgG1 and IgG2a - Histamine levels, MCP-1 levels - IL-4, IL-10 and IFN-γ expression - Histology	Restored temperature, ↓Clinical score ↓ IgE, IgG1 and IgG2a ↓ MCP-1 and Histamine ↓ Degranulation of mast cells in intestine ↓ IL-4, IL-5, IL-13 ↑ IFN-γ	(70)
9	Non-digestible short- and long-chain fructo-oligosaccharides	scFOS/lcFOS and scFOS/lcFOS with low dose OIT	Peanut extract	C3H/HeOuJ, i.g.	- Preventative and Treatment	- Symptomatic Scoring - mMCP-1 - PE-Specific IgE, IgA, IgG1, and IgG2a in serum - ELISPOT for peanut specific IgG1 and IgA specific lymphocytes	↓ Clinical score, Restored temperature ↑ IgG1, IgA and IgG2a	(71)
10	Non-digestible short- and long-chain fructo-oligosaccharides	- scFOS/lcFOS - SCIT	Peanut extract	C3H/HeOuJ, i.g.	- Preventative and Treatment	- Symptomatic Scoring - MCP-1 - Allergen-specific IgE, IgA, IgG1 and IgG2a levels in serum - IL-5, IL-10, IL-13 and IFN-γ	Restored temperature ↓ Clinical score ↑ IgE and IgG1 and IgG2a ↓ IL-5, IL-10, IL-13 and IFN-γ	(72)

*i.p., Intraperitoneal; i.g., Intragastric*.

#### Marine Algae

A wide variety of novel active metabolites from marine algae have been reported for their biological properties, in particular sulfated polysaccharides from various species of marine algae for their anti-inflammatory, anti-allergic, anti-coagulant and anti-oxidant activities (15, 60, 61, 73, 74). Three major groups of marine algae: brown algae, red algae, and green algae, are the main sources of sulfated polysaccharides. Several recent reports have indicated that chemically active metabolites from marine algae can suppress allergen specific antibodies such as IgE, IgG, IgG1 by downregulating CD3, CD4, and CD8 cell surface receptors thereby attenuating the cytokine response (e.g., IL-4, IL-5, and IL-13), in both *in vitro* and *in vivo* models of allergy (17, 75–78).

Fucoidan is a sulfated polysaccharide commonly found in different brown algae species. The anti-allergic activity of fucoidan from *Undaria pinnatifida* in ovalbumin-induced mouse airway hypersensitivity has been shown to suppress IL-4, IL-5, and IL-13 release and reduce concentrations of eosinophils in bronchoalveolar lavage (79). Subsequently, another study reported the role of fucoidan in reducing OVA-specific IgE levels by suppressing IgE specific B cell production; thereby downregulating IL-4 and upregulating IFN-γ expression levels in mice (Figure 2) (63). However, Yanase et al. also reported that intraperitoneal administration of fucoidan was more effective, as compared to orally treated mice, showing lower levels of OVA-specific IgE and IgG1 levels indicating the route of administration of fucoidans can influence allergic reactions (63).

In addition, alginic acid oligosaccharide (ALGO) from brown algae was shown to reduce serum IgE production in β-lactoglobulin susceptible mice (80). ALGO was also reported to suppress the Th-2 cytokine-based responses by downregulating IL-4 and upregulating IFN-γ and IL-12 production (Figure 2) (18, 80). Similarly, alginate extracted from *Laminaria japonica* demonstrated the prevention of allergic reactions in an OVA-induced allergy model by inhibiting mast cell degranulation and subsequent histamine release, lowering total serum IgE and IL-4 levels. Histological analyses showed improvement in the structure of intestinal epithelial villi, indicating a protective effect of alginate *in vivo* (81). In a passive as well as active cutaneous anaphylaxis model of ovalbumin and shrimp allergy in Balb/c mice, brown macroalgae extracts from *Sargassum tenerrimum, Sargassum cervicorne*, and *Sargassum graminifolium (turn)*, when injected peritoneally, suppressed the anaphylactic response (77). Based on these reports, intraperitoneal administration of polysaccharides seems to downregulate pro-inflammatory cytokines, IL-4, IL-5, and IL-13 levels, and suppressing the anaphylactic response through elevated production of IL-12 and IFN-γ. However, local administration of algal polysaccharides provides a significant challenge of extrapolating the required dose in humans which will be much higher as compared to the murine model (Figure 2). In a separate study, an *in-vitro* treatment using a methanolic extract from brown algae *Ecklonia cava* exhibited attenuation of histamine release from human basophil cells (KU81F cells), inhibiting IgE-FcεRI interaction to suppress degranulation and histamine release from basophil cells (78).

Porphyran, a sulfated polysaccharide isolated from red seaweed, popularly known as “Nori,” inhibited 2,4,6- trinitrochlorobenzene-induced contact hypersensitivity in mice through systemic downregulation of serum IgE antibody and IFN-γ levels, but no change was observed in local cytokines such as IL-4, IL-10, and IFN-γ when measured in ear lobes (76). Recently, Sacran, a sulfated polysaccharide from *Aphanothece sacrum* has been reported to suppress 2,4,-Dinitro-1-fluorobenzene-induced atopic dermatitis by ameliorating edema and restoring water content in the stratum corneum. The skin barrier function was markedly restored, while specific cytokines (IL1-β, TNF-α, and IFN-γ) and chemokine (MCP-1) mRNA levels were suppressed, indicating an overall anti-allergic effect in mice. Furthermore, *in vitro* administration of sacran to B cells isolated from spleen cells demonstrated no proliferative effect. However, *in vitro* sacran treatment in primary mouse T cells and Jurkat T cells significantly inhibited the proliferation of T cells, exhibiting immunomodulatory potential (17). Porphyran and sacran, as reported for contact hypersensitivity, could be further explored for their immune modulating properties for food allergy.

In red edible seaweed, specific polysaccharides such as Carrageenan, are reported to induce low dose antigen-dependent oral tolerance and lymphocyte anergy in β-lactoglobulin-fed C3H/HeJ mice. However, oral tolerance was only achieved in groups where β-lactoglobulin and carrageenan were fed before sensitization (75). Carrageenan on its own has been the center of controversy for its reported carcinogenic properties and toxicity in rodents (82).

Synbiotic formulations are prepared by mixing probiotic or prebiotics with functional dietary component and are reported for their efficacy in alleviating milk allergy in mice (20, 83–85). A mixture of probiotics with oligosaccharides have shown the ability to dampen allergic responses and alter the intestinal microbiota by reducing mast cell numbers and suppressing edema. Synbiotic formulations were also effective in reducing mast cell degranulation and alleviating anaphylactic symptoms (83, 84). Recently, dietary fructo-oligosaccharides have been reported to be involved in modulating oral sensitization to food allergens by attenuating CD4^+^ T cells and regulating Th-1 and Th-2 cytokine expression levels, thereby preventing OVA-induced food allergy (20). In a recent study, sulfated polysaccharides from red algae *Porphyra haitanensis* and *Gracilaria lemaneiformis* are reported to upregulate Treg cells and attenuate symptoms and cytokine release in an OVA-induced anaphylaxis model in mice (85). Although efficacious in preclinical studies, these synbiotic formulations are only reported for milk allergy. Further research using different allergy models should elucidate the mode of action and establishing possible translation to humans.

Functional components have also been derived from green algae and reported to be efficacious for a variety of diseases such as type-2 diabetes, colitis and hepatocellular carcinoma (86–92). Reported studies indicate sulfated polysaccharides to be the most active component of green algae by modulating gut microbiota, gene expression levels and carcinogenesis in various mouse models (86, 89). Flavonoids and polyphenols extracted from green algae have been shown to regulate gene expression and gut microflora in type-2 diabetes mouse models (87, 91). Furthermore, alkaloids are shown to attenuate colitis in murine models (88, 90). A recent systematic review on ulvan, a cell wall polysaccharide commonly found in green algae; explored various functional components, their toxicity and biological significance, indicating that ulvans may prove to be beneficial for preventing allergic diseases (47). In another study, it was reported that ulvan isolated from green algae *Ulva ohnoi* demonstrated no toxicity, and only mild immunomodulatory properties through elevated levels of IL-10 and decreased levels of prostaglandin E2 (PGE2) in lipopolysaccharide (LPS) stimulated murine macrophages (93). This study also reported that higher molecular weight components of the isolated fraction had elevated immunomodulatory response in LPS stimulated murine macrophages; however a minor elevation in pro-inflammatory cytokines such as IL-6 and IL-1β was also noted (93).

Currently, limited research evidence is available demonstrating the efficacy of green algae to attenuate allergic symptoms. However, some active functional components could be utilized in future for further investigations against food allergy.

#### Non-algal Polysaccharides

In addition to marine algae, mushrooms and yeasts are prime sources of non-digestible polysaccharides (58, 94). Compared to fucoidans or other sulfated polysaccharides, β-glucans modulate pro-inflammatory cytokines, such as TNF-α and IL-6 (58, 94–96). β-glucans also play a vital role in modulating Th-1-inducing cytokine responses including IFN-γ and IL-12. β-glucans might prove effective in alleviating food allergies, which are primarily a Th-2 biased response, by shifting the cytokine expression toward Th-1 cytokines, thus suppressing clinical symptoms including decreased body temperature and diarrhea (97). Generally, β-glucans are considered safe but some side effects have been reported, such as induction of nitric oxide by inducible nitric oxide synthase (iNOS) and in some cases the induction of inflammatory reactions (98).

Chitin and its derivatives are also reported to possess anti-allergic potential with low to no toxicity (99–101). Chitin, an abundant natural polymer with β1–4-linkages, is found in the exoskeleton of insects and crustaceans as well as in the cell walls of fungi. The deacetylated derivative is known as chitosan (102). According to Min-Jung Bae et al., mice fed with specialized diets consisting of chow mixed diet with α-chitin, β-chitin, and chitosan, reduced the production of allergen specific serum IgE in a peanut-induced anaphylactic mouse model. In all three chitin fed groups of mice, the Th-2 cytokine response was suppressed, leading to reduced levels of IL-5, IL-13, and IL-10 (101).

Inulin and oligosaccharides have been reported for being non-toxic and considered safe for human consumption (103). Human milk derived oligosaccharides mixed with inulin, have been reported to be effective in suppressing OVA-induced food allergy in mice (104). For instance, galacto-oligosaccharides and inulin supplementation (GOS/inulin) aid in preventing food allergy in mice offspring's by feeding GOS/inulin combination to the mother (105). The GOS/inulin combination altered the gut microbiota, providing long-term protection from food allergy in the offspring while dampening allergic symptoms and modulating the Th-1/Th-2 cytokine balance in the mother's immune system. However, no direct evidence was presented that the offspring's immune system was influenced in any way due to the administration of GOS/inulin to the mother only (105). In another study, human milk oligosaccharides, 2'-fucosyllactose and 6'-sialyllactose, were observed to suppress mast cell protease-1 expression and mast cell numbers, with elevated expression levels of Th-1 cytokines such as IL-10 and TNF-α in an OVA-sensitized mouse model. A significant decrease in mast cell numbers indicated downregulated OVA antigen reactivity but total IgE as well as OVA-specific IgE and IgG1 and cytokines (IL-6 and IL-13) remained similar or higher as compared to the OVA induced group (104).

All of the aforementioned studies present preliminary evidence on the efficacy of natural compounds from algal and non-algal sources in preclinical studies. However, most of these studies have inherent limitations related to compounds such as limited knowledge of *in vitro* cytotoxicity of compounds, and dose-response effect for desensitization. In addition, translating the treatment dose from mice to humans can be difficult, as what is considered a “safe dose” in mice might not be safe or efficacious in humans (106).

Various dietary polysaccharides, in addition to some prebiotic mixtures as discussed above, appear to elicit diverse immunomodulatory effects in different tissue sites, including the blood, gastrointestinal tract, MLN and spleen. The exact mechanism underlying the immunomodulatory activity of polysaccharides is not fully explored but based on existing reports the inhibitory effects of polysaccharides on allergic responses show a close association with immune-potentiating activity. However, most of the investigated models include only a narrow range of food allergens, providing the opportunity for further investigations on the anti-allergic potential of dietary polysaccharide for additional clinically relevant allergenic food sources.

### Traditional Chinese Medicine (TCM)

One of the most ancient and popular traditional medicinal practice systems is traditional Chinese medicine. TCM's are widely investigated in health research for developing new therapeutics for different diseases, including allergies, because of their reported effectiveness, low cost, and fewer side effects (16). However, the herbal medicinal system still lacks adequate studies demonstrating efficacy in food allergy treatment. Some herbal medicines are widely reported to be effective in several randomized, controlled trials of asthma (107–110), indicating that TCM has the potential as a natural therapeutic in alleviating food allergy symptoms. A summary of current research studies on TCM and other medicinal plants is provided in Table 2.

**Table 2:** A summary of studies investigating the immunomodulatory effects of traditional Chinese medicine and other medicinal plants, tested for preventative (prophylactic) or treatment (curative) strategies, using mouse models of food allergy.

**Sr. no**.	**Source of TCM**	**Component of TCM**	**Allergen source**	**Strain, route of exposure**	**Treatment strategy**	**Examined parameters**	**Outcomes**	**References**
1	*Actinidia arguta* (Hardy kiwifruit)	PG102	Ovalbumin	BALB/c, i.p.	- Preventative	- Levels of IgE, IL6, IL-10 - MCP-1, Mast cell infiltration	↓ Incidences of diarrhea ↓ IgE, IL-6 and MCP-1 in serum inhibited the infiltration of CD117 ^hi^ FcεRI ^hi^ mast cell	(111)
2	Multiple Chinese medicinal herbs	FAHF-2	Peanut	C3H/HeJ, i.g.	- Preventative	- Clinical symptoms - Histology scoring - Histamine levels - Peanut specific IgE and IgG2a levels - IL-4, IL-5, IL-13, IL-10, IFN-γ, and TGF-β	↓ Peanut-specific IgE ↓ Histamine ↓ IL-4, IL-5, IL-13 ↑ IgG2a and IFN-γ	(112)
3	Kakkonto	Commercially available Kakkonto	Ovalbumin	BALB/c, i.p.	- Preventative	- Clinical symptoms - Th-1-cytokines (IFN-g) - Th-2 cytokines (IL-4, IL-5, and IL-10) - mMCP-1 analysis	↓ Incidences of diarrhea ↓ IL-4 and IL-10 ↓ mMCP-1	(113)
4	*Panax ginseng*	Ginseng Saponins	Ovalbumin	BALB/c, i.p.	- Preventative	- OVA-specific IgG, IgG1, and IgA, IgE - IL-4, IL-12 and IFN-γ in activated splenocytes - Immunohistochemistry	↓ IgG, IgG1, IgA and IgE and cytokines but not significant	(114)
5	Apple Extract	Water extract containing polyphenols and flavonols	Ovalbumin	BALB/c, i.g.	- Preventative	- Allergy score - MMCP-1 - OVA-specific IgE, IgG1, and IgG2a - IL-4, IL-5, IL-10, and IFN-γ levels	↓ IL-4, IL-5, IL-10, IFN-γ	(115)
6	Multiple Chinese medicinal herbs	Commercial FAHF-2	Peanut, codfish and egg (multiple food allergy)	C3H/HeJ, i.g.	- Preventative and Treatment	- Symptom scoring - Histamine levels - IgE, IL-4, IL-5 IL-10, IL13, IFN-γ, and TGF-β levels	Symptomatic relief, ↓ Histamines ↓ allergen specific IgE ↑ IgG2a ↓ IL-4, IL-5, IL-10, and IL-13 ↑ IFN-γ	(116)
7.	Cissampelos sympodialis	Plant extract and alkaloids	Ovalbumin	BALB/c Wistar rats, i.p.	- Treatment	- Diarrhea scoring - OVA-specific IgE - IL-12p70, IL-13, and IFN-γ - Flow cytometry of mesenteric lymph node cells - Gut histology	↓Diarrhea incidences inhibition of mast and eosinophil cell activities ↑ proportion of Treg cells in the CD4+ T cell population	(117)
8.	*Arecae semen*	Polyphenol- enriched areca nut extracts (PANE)	Ovalbumin	BALB/c, i.p.	- - Preventative	- Diarrhea scoring - Spleen index and cellularity - Total IgE production - Histology - OVA-specific IgE	↓Diarrhea incidences, ↓ infiltration and degranulation of mast cells in the duodenum	(118)
9.	Formula-3	Mixture of 6 traditional Chinese herbs	Ovalbumin	Brown–Norway rats, i.g.	- Preventative and Treatment	- H&E staining of small intestine - Mast cell staining - IL-4, IL-10 levels - Histamine levels	↓ IL-4 and IL-10 ↓mast cell number in intestinal mucosa	(119)
10.	*Scutellaria baicalensis*	Baicalein	Ovalbumin	BALB/c, i.p.	- Preventative	- Diarrhea, temperature and anaphylactic score - OVA-specific IgE, IgG1, IgG2a level analysis - cytokine analysis (IL-4, IL-5, IL-10, IL-12, IL-13, IL-17, and IFN-γ)	↓Diarrhea score, reduction in anaphylactic response, restored temperature ↓IgE ↓ IL-4, IL-5, IL-10, IL-13, and IL-17	(120)
11.	Curcuma longa	Ethanol extract (dried) of Turmeric and curcumin	Ovalbumin	BALB/c, i.p.	- Preventative	- Diarrhea, temperature and anaphylactic score - OVA-specific IgE, IgG1, IgG2a, and mMCP-1 levels' - IL-4, IL-5, IL-13, IL-17 and TGF-β levels	↓ in diarrhea score, ↓ in anaphylactic response, restored temperature ↓ IgE ↓IL-4, IL-5, IL-13, and IL-17, ↑ TGF-β	(121)
12.	Poria cocos	Poria cocos bark (PCB) extract	Ovalbumin	BALB/c, i.p.	- Preventative	- Diarrhea - Temperature - Anaphylactic response - IL-4, IL-5, IL-10, IL-13, IL-17, TGF-β, and IFN-γ	↓Diarrhea occurrence, ↓ Anaphylactic response, restored temperature ↓IL-4, IL-5, IL-13 ↑ TGF-β	(122)
13.	*Scutellaria baicalensis*	Baicalein	Ovalbumin	BALB/c, i.p.	- Preventative	- Diarrhea - Temperature - Anaphylactic response - Total IgE - mMCP-1 levels - IFN-γ, IL-12, IL-4, IL-5, IL-13, IL-17, and TGF-β levels - Histological analysis	↓Diarrhea and anaphylactic response, restored temperature ↓ total IgE and mMCP-1 ↓IL-5, IL-10, IL-12, and IL-13 ↑ TGF-β	(123)
14.	*Citrus Tachibana*	Ethanol extracts of *C. tachibana* leaf extract (CLE), plant branch extract (CBE), and fruit body with peel extract (CFE)	Ovalbumin	BALB/c, i.p.	- Preventative	- Rectal temperature - Anaphylactic score - Diarrhea score - IL-4, IL-5, IL-12, IL-13, and IFN-γ levels	↓ Diarrhea and anaphylactic response, restored temperature ↓IL-4, IL-5, IL-13, IL-12, and IFN-γ	(124)

*i.p., Intraperitoneal; i.g., Intragastric*.

Studies investigating Chinese herbal medicine for the treatment of food allergy have used the following mixtures and components: food allergy herbal formula-1 (FAHF-1) (125), food allergy herbal formula-2 (FAHF-2) (126), Qian Cao (*Rubia cordifolia*) (113), and Qu Mai (*Dianthus superbus*) (127), and are discussed in more detail (113, 125–127). FAHF-1 and FAHF-2 in particular, are well-investigated as potential therapeutics for suppressing clinical symptoms, allergen specific IgE, IgG, and IgG1 antibodies, and associated cytokines such as IL-4, IL-5, IL-10, and IL-13 in murine models of peanut allergy (112, 125, 126, 128, 129).

A widely used 10-herb based formula, *Wu Mei Wan*, was combined with Ling Zhi (*Ganoderma Lucidum*) and named food allergy herbal formula-1 (FAHF-1) (125). FAHF-1 was reported to dampen anaphylactic symptoms by decreasing histamine levels in a mouse model of peanut-induced anaphylaxis. FAHF-1 alleviated the Th-2 cell response and reduced the levels of pro-inflammatory cytokines IL-4, IL-5, and IL-13, that are responsible for T-cell differentiation, activation of eosinophil, and basophil and goblet cell differentiation, leading to reduced lymphocyte proliferation in treated mice (125).

Upon further investigation, it was noted that two of the components in FAFH-1, Xi-Xin, and Zhi-Fu-Zi could be toxic if not prepared correctly. Therefore, these potentially harmful herbs were removed from the formula giving the updated 9-herb food allergy herbal formula-2 (FAHF-2) (130). Allergy treatment with FAFH-2 was tested in a murine model of peanut allergy, whereby mice displayed reduced anaphylactic symptoms after oral peanut challenge (126). In a subsequent study, the C3H/HeJ mouse model for peanut allergy was established, and FAHF-2 treated mice presented with no anaphylactic symptoms, and levels of peanut-specific IgE were reduced. (128). An increase in IgG2 levels was observed, accompanied by reduced levels of IL-4 and IL-5 in mesenteric lymph node (MLN) cells, leading to a Th-1 based response. An increased level of IFN-γ was also reported, showing modulation of intestinal Th-1 and Th-2 responses (112). There appears to be a synergistic effect of the FAFH-2 herbal mixture as the individual herbs failed to provide full benefit in a peanut induced anaphylaxis mouse model. A highly variable response of histamine levels, Th-2 cytokine such as IL-4, IL-5, and IFN-γ and peanut-specific IgE, IgG2a levels were reported when mice were treated with individual herbs from the mixture (129). Furthermore, in a clinical trial of double-blind, placebo-controlled oral food challenges (DBPCFCs) conducted in 68 patients with confirmed allergies ranging from peanut, sesame, fish or shellfish, FAFH-2 was shown to be safe as a herbal medication when confirmed using *in vitro* assays using patient PBMC's or by basophil activation; but the efficacy at chosen dose and duration showed no significant change in increase of tolerance to allergens when administered in allergic subjects (131).

Additional to the extensively studied FAFH-1 and FAFH-2 mixtures, *in vitro* studies of other TCM's have also been conducted. Lopez-Exposito et al. used the human B-cell cell line to investigate the anti-allergic potential of the medicinal herbs *Rubia cordifolia*, and *Dianthus superbus*, also known as Qian Cao and Qu Mai (127). The study demonstrated a decreased production of total IgE *in vitro* using U266 human B cells. Subsequently, lower peanut-specific IgE production and a suppressed anaphylactic response was observed in a peanut allergy-induced mouse model. The reduced levels of histamine production were also noted in the peanut challenge group, indicating a suppressed allergic response (127).

Overall, *in vivo* and *in vitro* cell-based studies suggest that TCMs that incorporate different natural herbs that were previously unexplored for their anti-allergic effects, have potential therapeutic value. However, as demonstrated by Wang et al. the use of FAFH-2 as a therapeutic medicine when investigated in a clinical study had no effect, indicating that proper dose selection and duration of the treatment are some of the limiting factors associated with translating TCM to therapeutic agents. Further research in elucidating the proper dose, toxicity and mode of action using humanized mouse model or *ex vivo* methods accompanying these natural herbs could be an essential step for future therapeutic applications.

### Other Medicinal Plants

Apart from the numerous studies using TCM, other medicinal plants and components of plants have been assessed in various preclinical and clinical studies of food allergy.

Kakkonto, a Japanese herbal medicinal mixture of seven herbs, is also reported to possess anti-allergic potential. Kakkonto was analyzed in a mouse model of OVA-induced food allergy to study changes in gastrointestinal symptoms (113) (Table 2). Kakkonto-treated mice demonstrated a lower incidence of diarrhea; however, no significant changes were observed for OVA-specific IgE compared to untreated mice. Differential expression and transcription analyses of proximal colon also revealed downregulation of the Th-2 cytokines IL-4, IL-5, IL-10, and IL-13, as well as Th-1 cytokine IFN-γ, upon Kakkonto administration. Histological analyses further indicated that the suppression was largely due to a decrease in mast cell numbers in the colon of the Kakkonto treated mice (113, 132, 133). In a separate study, Yamamoto et al. also demonstrated that Kakkonto administration effectively downregulated early antigen activation marker T cells (CD69^+^CD4^+^ T cells) and upregulated Foxp3^+^CD4^+^CD25^−^ T cells in the lamina propria, suggesting that Treg cells play a pivotal role in suppressing the effector T cell response (132). Furthermore, in another study these claims were supported by administering Kakkonto through oral immunotherapy with OVA and demonstrated upregulated Foxp3^+^CD4^+^ regulatory T cells (133). Kakkonto, despite showing promise in downregulating the expression of Th-2 cytokines locally in the tissue, was unable to reduce the antigen-specific antibody responses which could be a limiting factor if used as preventation alternative or in combination with oral immunotherapy.

A water-soluble extract, PG102 prepared from *Actinidia arguta* (Hardy Kiwi), also suppressed allergic diarrhea in an OVA-induced mouse model, by reducing IgE production and inhibiting mast cell infiltration in the large intestine. PG102 treated splenocytes expressed lower levels of IL-6 and MCP-1, ameliorating the allergic response (111).

Red Ginseng extract of *Panax ginseng* (red ginseng root) was also investigated in an OVA- induced mouse model (114). Mice treated with red ginseng extract displayed reduced IgG1 levels, however no change in OVA-specific IgE levels. The treatment nevertheless altered the expression levels of IL-12 and IFN-γ in stimulated splenocytes by upregulating the Th-1 response. Also, the extract-treated mice demonstrated elevated levels of CD8, IFN-γ, and IgA-positive cells in the small intestine, indicating suppression of the allergic response (114).

The efficacy of polyphenol-enriched apple extract was reported in an OVA-induced food allergy model (115). BALB/c mice fed with apple extract had significantly reduced mouse mast cell protease (MMCP-1) levels, thus suppressing allergic symptoms after challenge. Re-stimulated MLN cells had lower levels of IL-4, IL-5, and IL-10; 2-fold differences in relative mRNA gene expression of IL-5, IL-13, CCL11, IL-10, and IFN-γ cytokines in Peyer's patches as well as IL-13, CCL11, GATA3, IL-12, and IFN-γ in the ileum, indicated suppression of the local allergic response in the intestine. The findings indicate that protein–polyphenol interaction might be the mechanism behind mitigating the gene expression of cytokines and the production of mast cell proteases, thereby reducing the allergic response in mice (115).

*Cissampelos sympodialis (Bindweed)* and *Arecae semen (Areca Seed)* extracts have also been reported as anti-allergic in OVA-induced food allergy models. In two separate studies, *C. sympodialis* derived alkaloids, and *A. semen-*derived polyphenols decreased OVA-specific IgE and IL-4 cytokine levels. *A. semen* has also been reported to induce functional myeloid-derived suppressor cells, primarily responsible for the suppression of T-cell responses. Both extracts have been reported for attenuating the allergic response by increasing gut mucus production, thereby reducing antigen retrieval permeabilization and downregulating pro-inflammatory cytokine levels (117, 118). However, *A. semen* has also been reported for its toxic effects in rats if high doses are administered for extended times which does limits its efficacy if the dose has to be increased for humans (134).

Previous studies conducted on the anti-allergy effects of *Scutellaria baicalensis* (skullcap) extract, purified epicatechin (natural phenol and antioxidant) and polyphenol-enriched extract, *Curcumin longa (*turmeric), *Citrus tachibana* (tachibana orange) leaf extract and ethanolic extract of *Syzygium formosum*. Here, a modulated Th-1 immune response to counteract the antigen-specific Th-2 cell immune response in an OVA-induced mouse model were observed (120, 121, 124, 135, 136). Treatment groups demonstrated a reduced OVA-specific IgE response, downregulated mast cell function, and decrease in IL-4, IL-5, IL-10, and IL-13 levels (120, 124, 135, 136). Allergic symptoms in treated mice were also diminished, probably through the induction of Th-17 cytokines and IL-22, indicating an elevated Treg expression (120, 135). Despite showing promise as an anti-allergic *curcumin* the base compound isolated from *Curcumin longa* (turmeric) is now classified as a pan-assay interference compounds (PAINS) candidate (137). PAINS compounds are chemically classified compounds known to exhibit false positive results in bioassays and are screened using high throughput screening methods (138). The current body literature on PAINS is still emerging and adding more knowledge regarding the compounds that can potentially be interfering in the process of recognizing truly bioactive natural compounds (139).

Treg cells are important for maintaining gut homeostasis and therefore, generated through introducing bioactive compounds to suppress allergic symptoms (123, 132). One such study involved Baicalein, a natural flavonoid isolated from *Scutellaria baicalensis* commonly known as Chinese skullcap, has been reported to induce a Treg cell response. Baicalein was also demonstrated to modulate the intestinal barrier function by regulating tight junctions, in an OVA-induced mouse model. Baicalein reduced Th-2 cytokine levels (IL-4, IL-5, IL-10, and IL-13) and mast cell degranulation. Intestinal barrier function was restored by maintaining tight junction protein levels of claudin-1, Zonula occludens-1 and the junctional adhesion molecule. Induction of Treg expression induction, as verified through CD4^+^Foxp3^+^ T cell differentiation-based gene expression levels, indicated the involvement of the aryl hydrocarbon receptor. Baicalein acted as a potent agonist leading to suppression of the allergic response through induction of Tregs (123).

Further research aimed at isolating a single or combination of bioactive molecules may lead to a targeted therapeutic approach for treating allergic diseases. Studies reporting clinical interventions based on natural bioactive compounds from plants are very limited in terms of food allergy. Future in-depth investigation could help in establishing plant-derived natural compounds as potential alternatives for alleviating food-induced allergic reactions.

## Healthy Gut and Microbiota

The human intestine has various types of bacteria that adapt to the environment of the host's intestine, creating the intestinal microbiota. Diet and breast milk plays a significant role during infancy as they influence the bacterial microbiota in humans in the early stages of life, and any dysbiosis in microbiota may contribute to the development of allergy (140). The gut microbiota is in a constant state of flux dictated by our diet and lifestyle. Hence, probiotic formulations can be used as an alternative source of commensal bacterial organisms to alleviate the dysbiosis of microbiota in diseased conditions such as food allergy. Administration of probiotics can modulate the production of secondary metabolites such as SCFA's, and protective antibodies (IgA) to counteract the loss of intestinal immunity (141). Studies investigating the efficacy of probiotic formulations for different food allergies are discussed below.

### Probiotics

The role of intestinal microbiota in the development of the host immune system and the induction of immune tolerance has recently gained interest surrounding the consumption of natural dietary supplements such as probiotics. Probiotics are considered safe for human consumption if sold as dietary supplements according to the FDA, as they fall under the broad category of food items (142). Despite the marked rise in food allergies and intolerances, probiotics remain underutilized as a treatment despite an emerging body of research implicating the critical role of gut microbiota and their metabolites in the treatment of food allergies and intolerances.

The human gut typically harbors a diverse array of intestinal microbiota, which is dominated by four bacterial phyla: *Proteobacteria, Firmicutes, Bacteroidetes, and Actinobacteria* (143). During the digestion process, food proteins are hydrolyzed through biochemical processes involving stomach acid, bile duct juices and enzymes such as pancreatin and pepsin. These hydrolyzing processes results in varying sizes of food proteins being broken down into smaller peptides while some remain intact. These peptides and intact proteins are then subsequently presented to gut-associated lymphoid tissue (GALT) (144, 145). Early life exposure to smaller peptides from hydrolyzed proteins in the gut tend to aid oral tolerance induction (146). The mucosal immune system of the gut (GALT) continuously distinguishes non-allergenic food antigens from allergenic food antigens to establish oral tolerance to specific food antigens (144, 145). Commensal bacteria in the gut can aid this immune tolerance against food allergens by interacting with the mucosal immune system of the gut (GALT) (23). Immune tolerance seems to be primarily achieved during the early stages of life when the mucosal barrier and immune system are immature (26). Early experiments using germ-free mice demonstrated that impaired structure and function of CD4^+^CD25^+^Foxp3^+^ Treg cells within the mesenteric lymph nodes and Peyer's patches could be the key mediators for the induction of tolerance (147–149). Similarly, another study in germ-free mice demonstrated that early introduction of *Bacteroides fragilis* can reinstate the impaired GALT and induce tolerance during the neonatal period by inducing Treg cells via IL-10 instigated pathways (150). A number of studies have indicated that alterations in the diversity of commensal gut microbiota (dysbiosis) could lead to the development of food allergies or other diseases (23, 26, 28, 151).

Clinical interventions to analyze the effects of such alterations have been mostly performed in infants and children. Decreased abundance of *Lactobacilli* and an increased abundance of *Staphylococcus aureus* seem to be associated with egg and milk allergies in children (152). Recently, *S. aureus* was also implicated as bacterial allergen in allergic diseases that may exacerbate symptoms of asthma, atopic dermatitis and allergic rhinitis (153). Another study on infants showed that decreasing the levels of the genera *Lactobacillus* and *Bifidobacterium* made infants up to 2 months of age susceptible to the development of allergy to egg white, cow's milk, and inhalant allergens (154). In infants, oral administration of *L. salivarius, L. paracasei, B. animalis*, and *B. bifidum* significantly reduced the occurrence of atopic sensitization to common food allergens (155). This alteration in microbial diversity not only leads to an increased risk of food allergies but may also enhance the possibility of non-immunological food intolerances to gluten, and indigestible polysaccharides (Fermentable Oligo-, Di-, Mono-saccharides And Polyols, or FODMAPs) (156). Probiotic administration has been shown to significantly alter the gut microenvironment through promoting changes in the local microbial diversity, and holds therapeutic potential for the treatment of food allergy and induced intolerance (23).

Preclinical studies using probiotics have investigated the mechanisms leading to the amelioration of food allergic outcomes (Table 3). In an OVA-sensitized murine model, the introduction of *Bifidobacterium infantis* upregulated commensal gut bacteria including *Coprococcus* and *Rikenella* at the genus level, which are usually responsible for maintaining immune homeostasis. Subsequently, mice that were orally administered with probiotics were reported to have low levels of allergen-specific IgE and IgG1, suppressed diarrhea and lowered IL-4, IL-5, and IL-13 levels (Figure 3) (151). A recent study reported the role of commensal bacteria, colonized in germ-free mice from healthy infants and cow's milk allergic infants, exhibiting different transcriptome signatures in ileum measured using 32 differently expressed genes (28). This study also reported protection against cow's milk allergy in germ-free mice colonized with fecal matter from healthy infants, and reported *Anaerostipes caccae*, a clostridial species as being responsible for preventing food allergy development (28).

**Table 3:** A summary of pre-clinical studies investigating the role of probiotics tested for preventative (prophylactic) or treatment (curative) strategies, in reducing the progression or clinical symptoms in various mouse models of food allergy.

**Sr. no**.	**Source of probiotics**	**Bacteria (genera or strain)**	**Allergen source**	**Strain, route of exposure**	**Treatment strategy**	**Examined parameters**	**Outcomes**	**References**
1	VSL#3	*Lactobacillus acidophilus, L. delbrueckii* subsp. *bulgaricus, L. casei, L. plantarum, Bifidobacterium longum, B. infantis, B. breve, Streptococcus salivarius* subsp. *thermophilus*	Shrimp tropomyosin	C3H/HeJ, i.g.	- Treatment	- Clinical symptoms - Histamine (fecal) - Cytokines (IL-5, IL-10, IL-13, and IFN-γ) - FACS (anti-CD3 and anti-CD4) - Intracellular cytokine staining (IL-4, IL-10, IL-17, and IFN-γ) - Tropomyosin specific IgE, IgG1, and IgG2a - Total IgA (feces) - Tropomyosin specific IgA (feces) - Foxp3, IL-17, and IL-27 expression (jejunum) - IL-10, TGF-β, IL-4, IL-5, IL-13, and IFN-γ levels (jejunum extract)	↓ Clinical symptoms ↓ Histamine levels ↓ in tropomyosin-specific IgE ↑ in tropomyosin-specific IgG2a ↓ IL-5 and IL-13 ↑ IL-10 and IFN-γ ↑ IFN-γ^+^ and IL-10^+^ CD4^+^ T cells No change in IL-4^+^ T cells IL-17^+^ cells not induced ↑ in total IgA and tropomyosin-specific IgA ↓ IL-4, IL-5 and IL-13 ↑ FOXP3, IL-10, IL-17, IL-27, and TGF-β expression	(157)
2	*Lactobacillus brevis* HY7401, *L. casei* YIT9029 strain Shirota, *Bifidobacterium longum* HY8001	*Lactobacillus brevis, L. casei* and *Bifidobacterium longum*	Ovalbumin	OVA-TCR transgenic, i.g.	- Preventative	- Clinical symptoms - OVA-specific IgE, IgG1 and IgG2a - Total IgE, IgG1 and IgG2a - Cytokines (IL-4, IL-5, IL-6, IL10-, IL-12, and IFN-γ)	Mild relief in clinical symptoms ↓ in total IgE, IgG2a ↓ in OVA-specific IgE, IgG1 IgG2a ↑ IFN-γ ↓ IL-5, IL-5, IL-6, and IL-10	(158)
3	Purina Mouse Lab Diet 5015 supplemented with Primalac 454 Feed Grade Microbials	*L. acidophilus, Lactobacillus casei, Bifido bacterium bifidium, and Enterococcus faecium*	Peanut extract	C3H/HeJ, i.g.	- Preventative	- Body weight - Total plasma IgE levels - *L. acidophilus* quantity in feces - Flow cytometry for lymphocyte cells - PCR array for Th-1 and Th-2 cells gene expression	No observable clinical symptoms ↑ count of *L. acidophilus* No change in body weight of treated group ↑ IgE levels ↑ CD4^+^CD25^+^ splenic lymphocyte ↑ CD4^+^CD25^+^FoxP3^+^ positive Treg cells ↓ CCL11, IL-13, IL-6, IL-9, TNF-α and IL-17 expression	(30)
4	Extracellular vesicles or *B longum* KACC 91563 and *E faecalis* KACC 91532 mixture	*Bifidobacterium* and *E faecalis*	Ovalbumin	BALB/c, i.p.	- Preventative	- Clinical symptoms - Apoptosis assay - *In situ* TUNEL assay - Confocal microscopy - Proteomic analysis - Flow cytometry - ELISA for IL-4, IL-5, IL-10, IL-13, IL-17A, IFN-γ, and MCPT-1 - Ova-specific IgE ELISA	↓ in diarrheal occurrence upon feeding *B longum* KACC 91563 ↑ in diarrheal occurrence upon feeding *E faecalis* KACC 91532 No effect on OVA-specific IgE levels ↑ in IL-4, IL-5, IL-9, Il-10, and IL-13 IL-17 ND ↓ in MCPT-1 ↑ apoptosis by *B longum* KACC 91563 ↑ uptake of extracellular vesicles by BMMC's	(159)
5	*Lactobacillus, Bifidobacterium, Lactococcus, Streptococcus*	21 *Lactobacillus species*, 6 *Bifidobacterium species*, 2 *Lactococcus species*, and 2 *Streptococcus species strains*	β-Lactoglobulin	BALB/cByJ mice, i.g.	- Preventative	- Human PBMC's stimulation by probiotics - Total IgE, Total IgG1, Total IgG2a - BLG-specific IgE - Anti-BLG IgG1 and IgG2a - Plasma levels of MCP-1 - Cytokines (IFN-γ, IL-12p70, IL-4, IL-5, and IL-10) in BLG-stimulated splenocytes and MLN's - Th-1,Th-2,Th-17 and Treg gene expression - Cecal-microbiota analysis	↓ IL-4, IL-9, IL-17A, and IL-22 (PBMC's) ↑ IL-6, IL-10, IL-12p70, and IFN-γ (PBMC's)	(160)
6	Milk, Probiotic Dahi (La-Dahi and LaBb-Dahi) and Normal Dahi	*Lb. acidophilus* LaVK2, *Bifido. bifidum* BbVK3, *Lactococcus lactis* ssp. *cremoris* NCDC-86, and *Lc. lactis* ssp. *Lactis biovardiacetylactis* NCDC-60	Casein/Whey protein	Swiss albino, i.p.	- Preventative	- Whey protein specific IgA in intestinal fluid - Whey protein specific IgE in serum - IL-4, IL-10, IL-12, and IFN-γ - RT-PCR for IFN-γ, β-microglubulin, TGF-β, IL-4 and IL-10	↓ total IgE (La-Dahi and LaBb-Dahi) ↓ specific-IgE (LaBb-Dahi) ↓ IgG (La-Dahi and LaBb-Dahi) ↑ specific-IgA (Dahi, La-Dahi and LaBb-Dahi) ↓ IL-4 (Dahi, La-Dahi and LaBb-Dahi) ↑ IL-10, IFN-γ, IL-12 (Dahi, La-Dahi and LaBb-Dahi) ↑ IFN-γ and IL-10 expression (Dahi, La-Dahi and LaBb-Dahi) ↓ IL-4 expression (La-Dahi and LaBb-Dahi)	(161)
7	*Bifidobacterium infantis* CGMCC313-02 powder	*Bifidobacterium infantis*	β-Lactoglobulin	BALB/c, i.g.	- Preventative	- β-Lactoglobulin-specific IgE and IgG1 - Total IgE - IL-4, IL-10, IL-13, and IFN-γ in serum - Histology of intestine	↓ total IgE and IgG1 (prevention and pre-treated groups) ↑ Body weight ↓ signs of inflammation (prevention and pre-treatment groups histology) ↓ IL-4, IL-10 and IL-13	(162)
8	*Bifidobacterium infantis*	*Bifidobacterium infantis*	Ovalbumin	BALB/c, i.g.	- Preventative	- Ova-specific IgE and IgG1 - 16s rRNA sequencing - Bioinformatics analysis	↓ OVA-specific IgE and IgG1 ↓ IL-4, IL-5, and IL-13 Positive correlation between operational taxonomic units(OTU's) between groups Abundant *Bacteroidetes* and *Firmicutes* (Phyla level) Abundant *Lachnospiraceae* *S24-7, Rikenellaceae*, and *Ruminococcaceae* (Family level) Abundant *Coprococcus* and *Rikenella* (Genus level) 143 unique KEGG Orthologs (KOs)	(151)
9	Korean traditional fermented foods and Kimchi	*Lactobacillus pentosus* KF340*, Lactobacillus paracasei*698*, Lactococcus lactis* KF140*, Pediococcus pentosaceus* KF159 and *Bacillus subtilis* 26N	Ovalbumin	BALB/c, i.p.	- Preventative	- Clinical Symptoms - Total IgE - Ova-specific IgE - Cytokine levels (IFN-γ, IL-12, IL-4, IL-5, IL-13, IL-10, and IL-17) - Immunofluorescence staining (CD4^+^Foxp3^+^ lymphocytes)	↓ anaphylactic response ↓ diarrhea Restored temperature ↓ total IgE ↓ OVA-specific IgE ↑ IL-12 and IFN-γ ↓ IL-4, IL-5, IL-10, IL-13, and IL-17 ↑ CD4^+^Foxp3^+^ T-cell population and TGF-β	(163)
10	*Lactobacillus plantarum* ZDY2013, *L. plantarum* WLPL04, *L. rhamnosus* GG	*Lactobacillus plantarum, Lactobacillus. rhamnosus*	β-Lactoglobulin	BALB/c, i.p.	- Preventative	- Clinical symptoms - Total IgE, IFN-γ, IL-4, IL-17A, and TGF-β - Histology (colon) - IL-12A, IFN-γ, IL-4, IL-10, TNF-α, TBX21, GATA3, RORC, Foxp3, OCLN, CLDN1, and TJP1 expression in Ileum - 16s rRNA sequencing	↓ anaphylactic response ↓ total IgE ↑ IFN-γ ↓ IL-4 and TGF-β ↑ TBX21, IFN-γ, IL-12, IL-10, FOXP3 (LGG) ↓ GATA3, RORC, TBX21, IFN-γ (ZDY2013) ↓ IL-4 and GATA3 (WLP104) ↑ FOXP3 (WLP104) ↑ CLDN1 (LGG, ZDY2013) and OCLN (ZDY2013) Prevent histological changes (LGG, ZDY2013, WLP104) Abundant *Firmicutes, Bacteroidetes, Proteobacteria*, and *Actinobacteria* (phyla level) Significant taxonomic differences	(164)

i.p., Intraperitoneal; i.g., Intragastric

Disruption to the barrier function of intestinal epithelium increases the permeability of the mucosal lining, increasing exposure to allergens, pathogens, and toxins. Tight junction integrity plays a vital role in preventing antigen uptake from the intestinal lumen to the blood and is responsible for maintaining the structural integrity of the lumen. Probiotics are mainly comprised of *Lactobacillus, Bifidobacteria* and *Saccharomyces* genera which release metabolic molecules such as SCFA's, including butyrate, that up-regulates the expression of tight junction proteins (165).

Allergic disorders are the result of IgE antibody isotype switching by IL-4, and moderately by IL-13, leading to the upregulation of pro-inflammatory cytokines including IL-5, IL-6, and the subsequent allergen-specific IgE response. Such responses occur as a result of a shift in the Th-1/Th-2 balance that leads to an elevated Th-2 response. Probiotics can diversify the local microbiota and thus modulate immune dysbiosis by enhancing the Th-1 response (Figure 3) (166). A diversified local microbiota can potentially activate Toll-like receptors such as TLR-2 that are responsible for the differentiation of IgA from naïve B cells. In turn, increased IgA production can assist in the development of immune tolerance against IgE mediated allergic responses (167) (Figure 3).

Treg cells play a vital role in the acquired immune response by suppressing the action of mast cells and basophils, which results in downregulation of the IgE-mediated response. In a mouse model of OVA-induced food allergy, Treg-associated TGF-β production was induced by oral administration of *Lactobacillus acidophilus* strain L-92 (168).

These promising findings in a preclinical scenario indicate that probiotics can improve the homeostasis of intestinal microbiota and reduce intestinal inflammation. Most of the aforementioned studies have provided a promising outlook on the potential of probiotics, but more empirical evidence and clinical data is needed for recommending the use of probiotics against food allergy. Currently no official guidelines or clinical recommendations are in place for the use of probiotic formulations as a preventative or treatment regimen for food allergy. However, the current body of literature present a strong case for probiotics as a potential therapy in preclinical studies for food intolerances and allergic diseases.

Probiotic use in randomized clinical trials have resulted in mixed and inconsistent data showing little efficacy for preventing food allergy (169). Different systematic reviews and meta-analyses of randomized control studies, reporting on the use of probiotic formulations or specific bacterial strains and concluding that the current body of evidence has technical fallacies, biasness and imprecise estimated effects. The overall conclusion of these systematic reviews was that supplementing probiotics to infants, pregnant women or children does not reduced the risk of food allergy (169–171). However, more stringent and rigorous research approaches could be designed, based on already available studies, to identify probiotic supplementations that still holds a promising position to help individuals with food allergy.

## Current Pitfalls and Future Perspectives

Current research into natural products and probiotics has demonstrated potential beneficial effects on the human immune system and gut, with promising results in the prevention and treatment of various diseases. The current literature also demonstrates positive effects of these natural sources in suppressing allergic symptoms, mostly through reducing the generation of allergen-specific IgE antibodies, downregulation of effector cell activation (e.g., mast cells) or expression of Th-2 cytokines assisting in the progression of allergic reactions. However, the majority of investigations analyzing various natural sources have been conducted in small animal models or using *in vitro* cell-based assays. Most studies discussed in this review are lacking the assessment of *in vitro* cytotoxicity of the reported bioactive compounds or extracts before being investigated in preclinical studies (172). Another limiting factor affecting the design of these preclinical and clinical studies is that they lack PAINS compound screening as discussed earlier (138). Screening for PAINS compounds using electronic filters can limit false positive discoveries of certain compounds. However, PAINS filters should be implemented with utmost care as over simplistic approaches can lead to the selection of inactive compounds, which however can be addressed by following recommendations discussed by Baell et al. (139).

Screening for an alternate therapeutic approach is an important avenue and preclinical research serves as a crucial stepping-stone in the identification of potential therapeutic candidates for human use. It is essential that efficacy is investigated in a streamlined manner (e.g., *in vitro, in vivo* and phase 1 and 2 clinical trials) to reaffirm the safety and usefulness of such compounds. In general, mouse models that are currently being used for inducing food allergy have some intrinsic limitations. Presently, mouse models of food allergy are developed with the help of adjuvants (e.g., alum, freund's complete adjuvant and cholera toxin B) in genetically distinct mouse strains (e.g., Balb/c or C3H/HeOuJ) using different routes of sensitization (e.g., intraperitoneal and intragastric) (173–175). Hence, most studies reporting the efficacy in murine systems tend to fail or show no efficacy when replicated in clinical studies (106). Proper reporting of studies by following the ARRIVE guidelines set for reporting research conducted in animals can help overcome some of these issues that are plaguing the reproducibility and translation issues in preclinical research (176).

Probiotic formulations also have several limitations such as risk of sepsis, overstimulation of the immune system and microbial resistance (177). Regulatory standards on the use of probiotics by the FDA are very relaxed as selling the formulations as dietary supplements is considered safe. However, if they are to be sold as a drug, probiotics would then need to go through the rigorous FDA drug screening and approval process (142, 178). Due to these ambiguous regulations, probiotics remain to be sold as dietary supplements thereby increasing the possible risks discussed earlier.

AIT, primarily based on the whole extract, purified proteins or peptides, are designed to be very specific to the implicated food allergen source, for e.g., peanut allergens. However, natural product or probiotic-based approaches appear to reduce the symptoms and/or target specific pathways independent of the implicated food source. This is a major advantage of such type of approaches for two reasons, (a) a single therapeutic approach can target several different types of food allergens, a medical state which is observed in most allergy sufferers with multiple co-sensitization and allergy to different types of foods, and (b) the cost of research and development for such broad range therapeutics is much lower as compared to AIT, which has to be established and validated for every single source of an allergenic food.

A combinatorial approach using probiotics and peanut oral immunotherapy has successfully demonstrated long-lasting clinical effects and suppression of allergic responses (179). Such innovative strategies combining allergen-specific immunotherapy with natural bioactive compounds into a suitable dosage regimen may hold the potential to have a safe and effective treatment strategy for food allergies.

## Conclusion

Food allergy is an ever-increasing health issue that can significantly impact the socio-economic status, diet and well-being of affected individuals (1, 2). Currently, there is only one curative treatment in the form of OIT specifically for peanut allergy, with only palliative treatments available for other food allergies to relieve clinical symptoms. Most cases of food allergies to peanut, tree nuts, shellfish or fish, are not outgrown, frequently resulting in life-threatening reactions. There is an urgent need to develop novel treatment approaches to tackle the growing rate of food allergy worldwide.

Natural products are a rich source of compounds with therapeutic potential for a wide range of human health-related diseases. While a variety of natural product-derived lead compounds have entered clinical trials and are used for treating diseases such as cancer and microbial infections, none are currently cleared for use to treat allergic diseases. Currently, AIT remains the frontline treatment option that is heavily investigated and recently implemented in clinical studies to suppress or treat food allergies. Natural products or probiotics can be used either in combination or separately as preventative therapeutics if proven efficacious. Current research outlined in this review highlights mainly preclinical data obtained for various natural bioactive compounds and their immunomodulatory effects against food allergy. Future research needs to be directed toward translating these preclinical findings into clinical trials aimed at establishing the safety, efficacy and dosage regimen for natural product-based therapies.

## Author Contributions

KP, AL, and SK developed the concept for the review. KP and SK wrote the manuscript. AT and EJ contributed to the editing and contextual design. All figures were designed and generated using www.biorender.com. All authors contributed to the development of the manuscript and approved the final version.

## Conflict of Interest

The authors declare that the research was conducted in the absence of any commercial or financial relationships that could be construed as a potential conflict of interest.
